# Autotransplantation, Surgical Repositioning of Retained Canine, and Apical Filling of Two Incisors with Root Resorption

**DOI:** 10.22037/iej.v13i2.20097

**Published:** 2018

**Authors:** Norberto Juárez Broon, Cuauhtémoc Bello Hernández, Oscar Ivan Ruiz Montañez, Elizabeth Díaz Rosales, Jaime Padrón Santana, Alejandra Zulema Calderón Escamilla

**Affiliations:** a *University of Guadalajara, Affiliated to the Regional Military Hospital of Specialties. Mexican Army and Air Force, Guadalajara, México; *; b *Regional Military Hospital of Specialties, Mexican Army and Air Force, Guadalajara, México; *; c *Dental Specialties Unit, Mexican Army and Air Force, Naucalpan, México; *; d *Military Graduate School of Health, Mexican Army and Air Force, Naucalpan, México*

**Keywords:** Autotransplantation, Endodontics, Endodontic Surgery, Root Resorption, Surgical Repositioning

## Abstract

The purpose is to show the autotransplantation and surgical repositioning of a retained canine, and the apical filling of central and lateral resorbed incisors from a 12-year-old female patient, healthy and with clinical absence of left maxillary canine. Radiographically, the retained canine between the resorbed central and lateral incisors was observed. Root canal treatment of the canine was performed after 8 weeks; apical curettage and placement of bovine graft in inter-incisal zone was done after 4 months. During 6 months, orthodontic traction of the canine was carried out with no positive results, and 12 months after the autotransplantation, surgical repositioning was performed. Clinical-radiographic control at 30 days and 24 months showed absence of inflammation, restoration and integration of the tooth-supporting structures. Autotransplantation combined with surgical repositioning of the retained canine and the apical filling of two incisors achieved the harmonious, aesthetic, functional, dental and psychological preservation of the patient.

## Introduction

Autotransplantation is the relocation of a retained or erupted tooth to another place in the same person [1]. This viable surgical procedure offers many different benefits as in cases of agenesis of premolars or incisors, and traumas affecting anterior teeth [2], where other alternative treatments could be implants or dental prosthesis [3].

The most frequently performed autotransplantation is the one of the third molar to the alveolar zone of the first molar, and of the premolars to zone of the anterior teeth. The success rate for an autotransplantation procedure is approximately 94%, when the donor tooth has an incomplete root development (¼ to ¾ of formation), compared to the autotransplantation of the tooth with closed apex, which has a success rate of 84% [1]. It is important to consider that the proper management of the tissues during surgery and preparation of the recipient zone, as well as taking care of the patient take part in the survival of the autotransplantation.

Surgical repositioning is the intentional controlled extraction of a tooth and its reinsertion to the same alveolus with the purpose of performing the filling of a perforation or fractures of the third cervical, in those cases where surgery extrusion or crown lengthening are contraindicated, and also with the intention of realigning the tooth with its own alveolus [2].

Surgical repositioning is considered as an alternative treatment in cases where orthodontic movement for tooth repositioning is not viable or is even impossible. This procedure must be performed as less traumatic as possible, and the time of exposure of the tooth out of the alveolus must be controlled. A period lower than 18 min is recommended [1]. The purpose of the present case is to show the autotransplantation, the surgical repositioning of a retained canine, the apical filling of two resorbed incisors, and the interdisciplinary management with orthodontics and periodontics to achieve an aesthetic, functional dental harmony.

**Figure 1:**
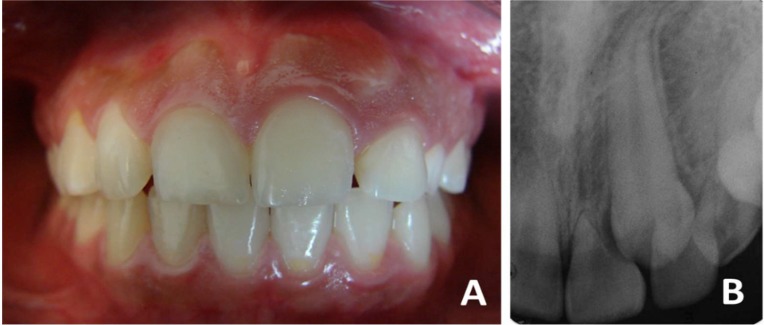
Clinical-radiographic appearance of retained canine and resorption in central and lateral incisors

**Figure 2:**
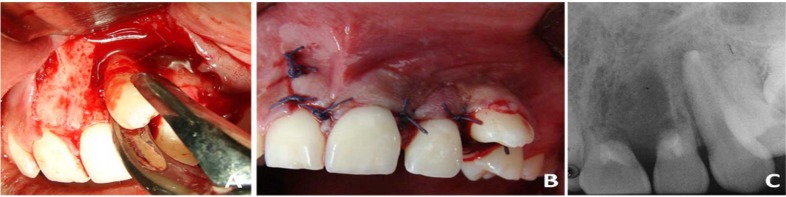
Extraction, autotransplantation of canine and apical filling of incisors

## Case Report

The female 12 year-old patient attended the Endodontic Department of the Regional Military Hospital of Specialties in Guadalajara, Jalisco, in appropriate general health conditions. The reason of consultation was a tooth that was still unerupted. In the clinical examination, presence of the left deciduous maxillary canine, absence of the permanent one, and increase of the gingival volume in the central and lateral left maxillary incisors area was observed (Figure 1A). In the initial periapical radiograph (E-Speed, Eastman Kodak, Rochester, NY, USA), retained left permanent maxillary canine in active phase of root resorption over the central and lateral incisors could be observed (Figure 1B). Retained canine and active root resorption of incisors was the diagnosis. The treatment plan included extraction of the deciduous canine and autotransplantation of the permanent canine to the prepared alveolar space of the deciduous canine. The rest of treatment was developed according to evolution of the canine and the incisors, as described below.

Clinical procedures were performed after local anesthesia with 2% lidocaine with 1:100000 epinephrine (Zeyco, Guadalajara, Jalisco, Mexico) and sterilized instruments. The deciduous canine was extracted *via* alveolar with #560 forceps (Safico, Vence Cedex, France). The recipient zone was prepared with a 2.8 mm diameter bur under abundant irrigation with 0.9% sodium chloride (CS, Baxter, Mexico). The permanent canine was extracted surgically with #65 forceps (Safico, Vence Cedex, France) (Figure 2A), after osteotomy with high speed handpiece (NSK, Japan), abundant water-spray irrigation, and surgical bur (Zekrya Endo burs, Dentsply, Maillefer, Ballaigues, Switzerland).

Apicoectomy, retro-instrumentation and retrograde filling with Super-EBA cement (Harry J. Bosworth, Skokie, IL, USA) was performed on the canine. The central and lateral incisors were retro-instrumented with K-type file #40 (Dentsply, Maillefer, Ballaigues, Switzerland) and filled with MTA cement (Angelus, Londrina, Paraná, Brazil).

The canine was stabilized on the previously prepared alveolus, though, in a vestibular position. The flap was repositioned and sutured with Vicryl (polyglactin) 3-0 (Ethicon, Edinburgh, UK) (Figure 2B). A final radiograph was obtained to verify the position of the autotransplanted canine and the apical filling of the incisors (Figure 2C). After 8 days, the suture was removed and the patient was maintained asymptomatic, with minor inflammation, absence of mobility of the canine, and with no postoperative complications. Root canal treatment on the canine was performed after 2 months, observing color change of the central maxillary incisor and collapse of the inter-incisal region; therefore, open curettage was performed, eliminating as well the granulomatous tissue with SG1/29LC curettes (Hu-Friedy manufacturing Co Inc., Chicago, Illinois, USA). With ED10 ultrasonic tip (Xpedent, England) under abundant irrigation with 0.9 % sodium chloride (CS, Baxter, Mexico), retro-preparation of the apical zone of both incisors was performed, and they were filled with Super-EBA cement, placing hydroxyapatite graft (Osteograf, CeraMed Dental, LLC, Lakewood, USA), which was confirmed with a periapical radiograph. It was sutured with Vicryl 3-0 and the same postoperative protocol was followed.

**Figure 3:**
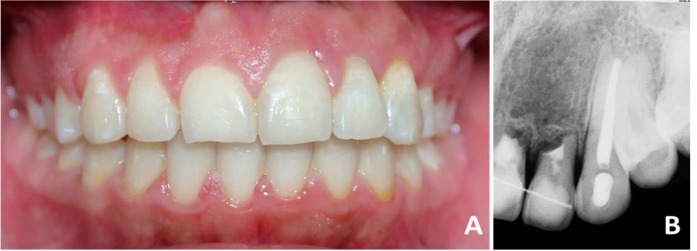
Braces removal and clinical-radiographic control after 2 years of surgical repositioning

After 2 months, corrective orthodontic traction and integration of the dental arch was initiated, using braces and 0.14, 0.16 and 0.18 archwires (3M Unitek, Monrovia, California, USA), which was applied for 4 months. With the phase 1 of the orthodontic treatment, 2 mm of mesiodistal space was obtained to place the canine in the dental arch. Owing to the fact that the canine was vestibularized, the traction with elastic band (American Orthodontics, USA) was attached to a vestibular button (Paratherm, Dentaurum, Germany), and open-coil spring (Paratherm, Dentaurum, Germany). The forced applied was of 50 mg, and for 30 days it didn't show movement of the canine, thus, it was decided to apply traction with a 16×22 transpalatal arch (Paratherm, Dentaurum, Germany) with extension towards the palatal surface of the tooth, and traction with 0.36 wire (Paratherm, Dentaurum, Germany) with a force of 50 mg was applied. In this way, no force or movements were exerted to the anterior incisors due root resorption.

After 6 months of orthodontic traction, 2.5 mm of mesiodistal space were obtained; however, it was not attained to include the canine in the arch, therefore it was decided to perform surgical repositioning. The full thickness mucoperiosteal flap was elevated with 2 releasing incisions between the maxillary lateral incisor and second premolar. The extraction of the canine was made with forceps #65, which was maintained hydrated with 10 mL of 0.9% sodium chloride (CS, Baxter, Mexico) for 5 min; in the meantime, the alveolus was prepared to approximately a 3.5 mm depth with 2.8 mm diameter bur and electric motor (Straumann Dental Implant System, Waldenberg, Switzerland) under an abundant irrigation with 0.9 % sodium chloride.

The canine was repositioned in the prepared alveolus, and a hydroxyapatite graft was placed (Osteograf N-300, Dentsply Ceramed, Lakewood, Colo, USA). The flap was sutured without complications. To avoid mobility and increase stability, the canine was fixed with 0.10 wire ligature from the lateral incisor to the maxillary premolar, a periapical radiograph was obtained, and the previously mentioned postoperative protocol was prescribed. Suture stitches were removed after 8 days. The patient was found asymptomatic, and the canine and incisors in appropriate conditions, with no mobility.

After 30 days, gingiva in phase of healing, minor inflammation, minimum dental mobility, harmony with soft tissues, and good overall state was observed in the clinical examination. Radiographically, apical filling of the incisors in good conditions and no periapical resorption of the canine was detected.

After 2 years, braces were removed and incisors were maintained with fixed retainer. Clinical absence of gingival inflammation, good tissue integration, minimum gingival retraction on the central incisor, absence of periodontal pocket, and good integration to the dental arch was observed. Radiographically, there is visible a hard layer around the canine, ankylosis on incisors, absence of periapical pathology, bone restoration in inter-incisal zone, and integration of bone and dental structures (Figures 3A and 3B).

## Discussion

Autotransplantation was demonstrated to be a potential treatment for tooth replacement, in case of trauma, agenesis or premature loss of a tooth caused by caries. It is a viable option for young patients and for those who don't have another clinical alternative; it is a reliable technique that has a success percentage of 74-100% [4, 5]. In a recent study of autotransplantation it was reported a success of 81% for 215 patients [6]. In the present case it was treated a maxillary canine (#23) in ectopic position, which was autotransplanted to the place occupied by the deciduous canine (#63) (previously extracted), since it caused root resorption of the central and lateral incisors (#21 and #22), and that were filled in a retrograde way with Super-EBA cement.

Root resorption of the incisors (#21 and #22) was initially sealed with MTA; nonetheless, it showed contamination produced by marginal apical leakage due to deficient hardening of MTA cement. A reason why MTA didn't harden and was contaminated could be that the surgical procedure, when the cavity was flooded with blood, caused leakage because of its prolonged time of hardening (2 h and 45 min) [7]. Hence, in a second surgery it was decided to perform a retrograde filling on the incisors with Super-EBA cement. It was demonstrated in other works that leakage of MTA was reduced by diminishing the time of hardening due to the addition of 10% calcium chloride to the MTA [8, 9].

There are two options to manage retained teeth: orthodontic and surgical; notwithstanding, treatment options depend on the retaining position (vestibular or palatal), its severity, and the age of the patient [3]. Dental autotransplantation is the first option for teenagers, since the usage of dental implants and prosthesis are not recommended in these patients, which are still in a stage of skeletal, dental and facial growth and development [1, 10]. One of the options is surgical management followed by orthodontic traction, in which the fixed appliances offer an alternative with the employment of elastic chain or band. However, in the present case, by resorting to orthodontic treatment, the traction movement (force of 50 mg) applied to the maxillary canine was not successful, because of the lack of anchorage from the incisors, reason why it was resorted to surgical repositioning of the maxillary canine performed in a third surgery, which allowed to achieve aesthetic and functional harmony in the maxillary dental arch.

Root canals treatment of the maxillary canine was performed 2 months after the first surgical procedure, since it was located in an accessible way for treatment; nevertheless, another study mentions that, as long as the tooth is in viable position for the root canal treatment, it can be done before the autotransplantation surgery, otherwise it has to be performed from 1 to 2 weeks after [1].

Autotransplantation and surgical repositioning entails certain risks, if appropriate management of surgical techniques is disregarded, such as necrosis and root resorption, consequently, the loss of the tooth; the aforementioned can be avoided if the asepsis conditions at the moment of surgery and the minimum time control outside the alveolus are maintained [2]. In the present case, the asepsis protocols of the surgery area were observed, and the maxillary canine was 5 min outside the alveolus when the autotransplantation and the surgical repositioning were respectively performed; accordingly, there was no root resorption after removing the fixed appliance. In regard to the ankylosis, it was present on the canine; notwithstanding, in another study on 12 anterior repositioned teeth, they shown firm retention in the alveolus and absence of ankylosis [11]; therefore, and despite of the likely ankylosis, the surgical management performed on the patient brought back dental aesthetic and functionality. 

## Conclusion

Autotransplantation and surgical repositioning of the maxillary canine, combined with filling of the central and lateral incisors, permitted the conservation of aesthetic and functional harmony, preserving the teeth, free from mobility, resorption, and periodontal disease.
